# Optimization of the Search for Neuroprotectors among Bioflavonoids

**DOI:** 10.3390/ph17070877

**Published:** 2024-07-03

**Authors:** Igor Belenichev, Victor Ryzhenko, Olena Popazova, Nina Bukhtiyarova, Nadia Gorchakova, Valentyn Oksenych, Oleksandr Kamyshnyi

**Affiliations:** 1 Department of Pharmacology and Medical Formulation with Course of Normal Physiology, Zaporizhzhia State Medical and Pharmaceutical University, 69000 Zaporizhzhia, Ukraine; 2 Department of Medical and Pharmaceutical Informatics and Advanced Technologies, Zaporizhzhia State Medical and Pharmaceutical University, 69000 Zaporizhzhia, Ukraine; 3 Department of Histology, Cytology and Embryology, Zaporizhzhia State Medical and Pharmaceutical University, 69000 Zaporizhzhia, Ukraine; 4 Department of Clinical Laboratory Diagnostics, Zaporizhzhia State Medical and Pharmaceutical University, 69000 Zaporizhzhia, Ukraine; 5 Department of Pharmacology, Bogomolets National Medical University, 01601 Kyiv, Ukraine; 6 Broegelmann Research Laboratory, Department of Clinical Science, University of Bergen, 5020 Bergen, Norway; 7 Department of Microbiology, Virology and Immunology, I. Horbachevsky Ternopil State Medical University, 46001 Ternopil, Ukraine; kamyshnyi_om@tdmu.edu.ua

**Keywords:** information technologies, multiple sclerosis, virtual screening, bioflavonoids, neuroprotection

## Abstract

For the first time, to optimize the creation of new neuroprotective agents based on bioflavonoids, we applied information technologies; these include docking analysis to calculate the binding of candidate molecules to the pharmacological target protein transthyretin as well as a program of virtual screening of NO scavengers. As a result of this approach, the substance catechin was isolated from candidate molecules—quercetin, catechin, Epicatechin gallate, Epicatechin, Procyanidin B1, Procyanidin B2, Procyanidin B3, and Catechin-3-gallate—according to docking analysis. As a result of virtual screening, catechin was identified as a potential NO scavenger (55.15% prediction). The results of the prediction were confirmed by in vitro experiments. Course administration of catechin to animals with experimental multiple sclerosis (MS) against the background of methylprednisolone administration completely eliminated lethal cases, reduced the number of diseased animals by 20% as well as prevented the development of severe neurological symptoms by 20% (compared to the methylprednisolone group) and by 60% compared to the control group. Course administration of catechin with methylprednisolone leads to a decrease in the neurodegradation markers in the cytosol of rats, with EAE: NSE by 37% and S-100 by 54.8%. The combined administration of methylprednisolone significantly exceeds the combination of methylprednisolone with the reference drug mexidol by the degree of NSE reduction. The obtained results indicate a significant neuroprotective effect of ocular combinations of methylprednisolone and catechin. The above-mentioned confirms the correctness of the bioflavonoid selection with the help of a virtual screening program.

## 1. Introduction

Multiple sclerosis (MS) is a progressive autoimmune disease of the central nervous system (CNS). A key aspect of MS pathology is the infiltration of brain tissue by T cells that cross the blood–brain barrier, leading to characteristic inflammation and demyelination [1]. This perivascular infiltration of inflammatory cells into the CNS is facilitated by their adhesion to endothelial cells and subsequent migration across the blood–brain barrier [2]. Proinflammatory cytokines synthesized by T cells—including interleukin-2, lymphotoxin, γ-interferon, and tumor necrosis factor-alpha (TNF-α)—contribute to a prolonged increase in blood–brain barrier permeability [3]. Additionally, these cytokines trigger the activation of microglial cells, macrophages, and astrocytes. In response, microglial cells secrete inflammatory cytokines and increased amounts of free radicals, promoting cell necrosis, membrane lysis, and significant damage to myelin and oligodendrocytes [4,5].

Conformational changes in human proteins also play a role in the pathogenesis of multiple sclerosis (MS). While the nature of these conformational changes is largely unknown, some lead to the formation of amyloid fibrils. These amyloid fibrils accumulate in the extracellular space of tissues, causing organ dysfunction [6,7]. MS is characterized by four main pathological features: Firstly, immunological changes in MS are considered to be due to an increase in the infiltration of proinflammatory cells, which consist of CD4+ T cells with T helper 1 (Th1)/Th17 phenotypes, B cells, monocytes, macrophages, and natural killer (NK) cells. There is also a decrease in the number of CD8+ T cells, CD4+ CD25+ forkhead box P3 (FoxP3+) Treg cells, and Treg 3 dysfunction. Secondly, the inflammatory process results in the destruction of the myelin sheath or the oligodendrocyte cell body, leading to demyelination. Thirdly, inflammation also causes axonal damage and loss. Fourthly, the astrocytic response to inflammation-induced neuronal damage, known as “gliosis”, occurs [6,7]. The primary target in multiple sclerosis is the myelin sheath of the CNS (both gray and white matter). The release of pro-inflammatory cytokines—such as interleukin 17 (IL-17), IL-4, IL-10, IL-12, IL-23, and TNF-α—along with the activation of macrophages and microglia, damages the myelin sheath of neurons. This damage is mediated through the secretion of toxic substances like reactive oxygen species (ROS), reactive nitrogen species (RNS), and glutamate [8,9]. 

Inducible nitric oxide synthase (iNOS) is recognized for its association with the progression of various autoimmune diseases. Inducible nitric oxide synthase (iNOS) is expressed on monocytes, which are crucial in initiating and developing the immune response. The induction of the enzyme is influenced by pro-inflammatory cytokines, immunomodulatory peptides, and even beta-endorphin through a mechanism involving an increase in cAMP [10,11,12,13]. Excessive nitric oxide (NO) production is associated with lesions observed in multiple sclerosis (MS). Transthyretin (TTR) is a plasma protein implicated in three amyloid diseases: familial amyloidotic polyneuropathy, familial amyloid cardiomyopathy, and senile systemic amyloidosis [14,15]. Senile systemic amyloidosis results from conformational changes in the wild-type protein, whereas the other two diseases are caused by gene mutations.

The crystal structures of wild-type TTR and numerous disease-causing mutants have been identified and studied in patients with multiple sclerosis (MS). Evidence suggests that certain natural antioxidants influence transthyretin expression [8,9,16,17,18,19,20,21]. The main classes of these antioxidants include phenolic acids, flavonoids, stilbenes, and lignans. These natural compounds possess strong antioxidant properties, which are frequently linked to their neuroprotective effects against various neurodegenerative diseases, including Alzheimer’s disease, Parkinson’s disease, Huntington’s disease, amyotrophic lateral sclerosis, and multiple sclerosis [22,23]. 

Various studies have demonstrated that certain polyphenols can inhibit the self-assembly of specific peptides and proteins associated with amyloid diseases. The formation of amyloid fibril aggregates contributes to several degenerative human diseases, initiating with protein dimerization and small oligomer formation. These structures promote the subsequent growth of protofibrils and fibrils, which can accumulate abnormally in tissues and organs, characteristic of these pathological conditions. Numerous small chemical compounds that interact with TTR have been identified. Both natural and synthetic ligands from diverse chemical groups have been studied for their ability to stabilize the TTR tetramer. Research has focused on understanding how structural modifications affect their efficacy and selectivity, either enhancing or diminishing their activity. Subsequently, numerous additional natural compounds have been examined for their potential to inhibit TTR fibril formation. These compounds have been crystallized in a complex with TTR, contributing to an expanded understanding of their interactions and binding affinity.

Researchers are highly interested in flavonoids as potential medicinal compounds because they exhibit a diverse array of pharmacological effects, including antimicrobial, anti-inflammatory, anti-aggregation, anti-amyloid, and antioxidant properties. Notably, many flavonoids can benefit the central nervous system by crossing the blood–brain barrier, mitigating oxidative stress, normalizing carbohydrate metabolism, and regulating the nitric oxide (NO) system [24].

In recent years, flavonoids—a group of natural antioxidants—have attracted attention for their potential neuroprotective properties against a range of neurodegenerative disorders. Research has highlighted that flavonoids exhibit varying abilities to bind to both thyroxine binding sites (T4-BS) of TTR, thereby inhibiting TTR amyloid formation through stabilization of the TTR tetramer [25]. The literature has published a variety of crystal structures of wild-type transthyretin (wt-TTR) in complex with various flavonoids, enabling researchers to examine TTR’s interactions with these flavonoids as well as the impact of hydroxyl group location and quantity on ligand affinity. The primary polyphenolic component of green tea, epigallocatechin gallate (EGCG), is the most well-known flavonoid. EGCG has a variety of biological properties, including anti-inflammatory and antioxidant properties. Furthermore, in vitro tests have demonstrated the strong anti-cancer properties of EGCG. For stopping the advancement of neurodegenerative illnesses like Parkinson’s disease, multiple sclerosis, Alzheimer’s disease, and amyotrophic lateral sclerosis, EGCG is seen to be a promising treatment option [26]. 

A class of naturally occurring substances called flavonoids is used, for instance, to treat vascular endothelial damage, having a reputation for being superior oxygen free radical scavengers. Since the nitric oxide radical (NO) is probably involved in this pathology, it has been established that flavonoids have the capacity to scavenge NO. It was discovered that flavonoids are highly effective NO scavengers. Anthocyanidins were identified as more efficient scavengers than hydroxyethylrutosides, which correlates with their therapeutic activity [27]. The values of their scavenging rate constants are only 30 times less active than those of the highly powerful endogenous hemoglobin scavenger of NO. It is assumed that NO removal contributes to the therapeutic effect of flavonoids [28]. The results obtained from crystal structure analysis are often combined with various physicochemical assessments in vitro or in vivo, making flavonoids very promising for the development of neuroprotective MS treatments.

The aim of this study. To identify the most active bioflavonoid with the properties of a TTR inhibitor and NO scavenger and to evaluate its effect in vitro and in a model of multiple sclerosis using modern information technologies (docking analysis, virtual screening program).

## 2. Results and Discussion

As can be seen from the docking analysis data presented in Table 1, Table 2, Table 3, Table 4, Table 5 and Table 6, L-thyroxine has a binding energy of −6.4 cal/mol for the specific binding sites of human transthyretin. Quercetin and catechin are comparable to L-thyroxine in terms of binding energy and amount to −6.7 cal/mol. According to the docking analysis, the test samples—Epicatechin gallate, Epicatechin, Procyanidin B1, Procyanidin B2, Procyanidin B3, Catechin-3-gallate—have a binding energy to specific sites of human transthyretin in the range of 7–8 kcal/mol. The obtained results theoretically and mathematically substantiate the prospects for further in vitro studies of catechin. 

As can be seen from the data presented in Table 2, in vitro experiments have shown that the addition of catechin to the incubation mixture leads to its binding to the protein transthyretin and displacement of thyroxine from the Transthyretin–L-thyroxine complex. This fact is evidenced by an increase in the optical density at 280 nm (formation of the catechin–transthyretin complex) and an increase in the optical density at 225 nm (displacement of -L-thyroxine from the complex and the appearance of its concentration in the incubation mixture). The results fully confirm the data of the docking analysis and indicate a high binding energy of this test sample with specific transthyretin binding sites.

Our results do not contradict the data of other researchers who have conducted a detailed analysis of ligand interactions for complexes with human TTR. There are two known thyroxine binding sites of TTR (TBS) located in the dimer–dimer interface between the inner sheets of two TTR dimers [29]. These sites have a funnel-shaped morphology with polar residues placed at the entrance and at the bottom and a hydrophobic core located at the center. Three small depressions in the TTR sites were found, which are responsible for the placement of T4. TTR sites can work with several classes of chemicals, including hormones and hormone analogs. There is evidence of flavonoid binding to TTR sites [30].

Despite the fact that catechin and quercetin had comparable docking analysis results, we chose catechin. Catechin and its metabolites are more active than quercetin in the regulation of Il-1b-dependent ROS production [31], it has a higher antioxidant activity [32], and a more pronounced attenuating effect on the genes IL-1β, TNF-α, and p53 [33].

When testing the structure of catechin using a virtual screening program, a result was obtained that predicted catechin’s ability to bind NO at 55.15%. Further testing of catechin using in vitro experiments within the system of photoinduced auto-oxidation of sodium nitroprusside and NO release revealed that at a concentration of 10–6 M, catechin inhibits the formation of radicals by 43.67%. Reactive oxygen and nitrogen species (RONS) are the result of regular cellular metabolism and include reactive nitrogen species (RNS) and reactive oxygen species (ROS). They can cause irreversible changes in the function of various essential macromolecules, such as proteins, membrane lipids, and nucleic acids, which can ultimately result in cell death. Both the onset and progression of brain injury as well as the pathophysiology of cognitive impairment are significantly influenced by oxidative stress. Many clinical situations, including neurodegenerative disorders, inflammation, and ischemia, are associated with increased nitric oxide (NO) production and heightened nitrosative/oxidative stress. The synthesis of NO in different cells is carried out by the enzyme nitric oxide synthase (NOS), which changes L-arginine into L-citrulline [10].

The NO signaling pathway is, therefore, a promising target for therapy. The NO signaling system is impacted by naturally occurring polyphenols, and this may have implications for neurodegeneration and its aftereffects. The peripheral and central nervous systems both use NO for a number of critical functions. It contributes to both neurotoxicity and neuroprotection. The vascular endothelium—which controls blood flow, lowers neuronal apoptosis, and prevents platelet aggregation—has been found to contain eNOS. Several investigations, a small number of which come from human brain autopsies, have shown that NO plays a major role in neurodegenerative illnesses. Reduced NO levels in endothelial cells are essential for elevating Aβ expression and influencing the amyloid precursor protein (APP) in cerebral blood vessels [10]. Widespread nitration of Lewy bodies and Lewy nitrite has been demonstrated in autopsy cortices of patients with Lewy bodies and Alzheimer’s disease. In addition, in patients with neurodegeneration with iron accumulation in the brain, nitration of α-synucleins in the glial cells of the exposed white matter of the cerebellum occurred; in patients with multiple systemic atrophy, as well as nitration of Lewy bodies and neuroaxonal spheroids, pale globe type 1 was detected [34]. 

A widespread prevalence of nitrated tau proteins in autopsy of the brains of MS patients has been demonstrated. The involvement of NO and its reaction product (with superoxide radicals), peroxynitrite, in MS pathology has also been reported in postmortem brain studies as exceptionally elevated levels of protein nitration were found in the hippocampus of MS patients compared to age-matched controls [10]. Several studies based on animal models of MS have shown that NOS inhibition slows the progression of disease pathology. The involvement of NO and peroxynitrite has also been reported in postmortem studies of MS-affected brains, where increased nitration of tyrosine residues in degenerating neurons of the compact part of the substantia nigra was reported [10]. In addition, nitration of protein tyrosine residues has become a crucial factor in the pathogenesis of a wide range of neurodegenerative diseases [35]. 

It is also interesting to note that most NO-mediated neurodegenerative pathogenesis occurs through nitration. Therefore, utilizing new information technologies, we have identified catechin from a vast array of bioflavonoids. According to the results of docking analysis, catechin can form complexes with transthyretin, a protein involved in the pathogenesis of multiple sclerosis (MS). Additionally, it can serve as a potential scavenger of NO, which also contributes to the pathogenesis of MS. Consequently, a computer program for virtual screening yielded a 55% prediction. In vitro studies have subsequently confirmed the high potential of catechin as a neuroprotective agent for the treatment of MS.

The results of our studies indicate that during the induction of EAE in animals of the control group, the development of neurological disorders of varying severity was recorded. Death was recorded for two rats (20% mortality). Inflammatory reactions were recorded at the injection site, which were observed throughout the experiment. It was recorded that after the formation of EAE in experimental animals, the appearance of persistent neurological disorders was noticeable on day 9. The maximum number of neurological disorders was recorded on day 14 of the experiment. The duration of time for the maximum effect was 4 days. The duration of EAE was 16 days. The average cumulative index was 27 points. At the peak of clinical manifestations of pathology, 30% of the rats had a clinical index of 0.5–2.5 points (mild degree of pathology); in 70% of the rats, the clinical index was from 3 to 6 points (severe pathology). The administration of methylprednisolone increased the survival rate of rats with EAE to 100% and reduced the number of animals with severe symptoms by 18%. When methylprednisolone was administered, the neurological disorders of EAE “at the peak” were shorter (*p* < 0.05) and in a milder form (*p* < 0.05).

The administration of catechin alongside methylprednisolone resulted in the complete elimination of fatalities, a 20% reduction in the number of sick animals, and prevented the development of severe neurological symptoms by 20% (in comparison to the methylprednisolone group) and by 60% compared to the control group. Additionally, neurological symptoms of experimental autoimmune encephalomyelitis (EAE) were shorter (*p* < 0.05) and manifested in mild or moderate forms with the administration of additional catechin. The clinical index at the peak of the disease decreased (*p* < 0.05), as did the cumulative index (*p* < 0.05). Therefore, due to its properties of binding to transthyretin and inhibiting NO production, catechin enhances the effect of methylprednisolone in EAE.

Similarly, the administration of the reference antioxidant drug Mexidol alongside methylprednisolone also resulted in the elimination of fatalities in rats with citicoline-induced EAE, along with the prevention of severe neurological disorders by 10% compared to the methylprednisolone group and by 50% compared to the control group. At the same time, the neurological symptoms of experimental autoimmune encephalomyelitis (EAE) were shorter (*p* < 0.05) and manifested in mild or moderate–severe form. Both the clinical index at the peak of the disease and the cumulative index decreased significantly (*p* < 0.05) compared with the control group; however, the additional administration of Mexidol with methylprednisolone did not lead to a significant increase in the therapeutic effect of methylprednisolone. Thus, unlike Mexidol, catechin is capable of significantly enhancing the effectiveness of methylprednisolone in EAE, which serves as an experimental equivalent of multiple sclerosis (MS).

As can be seen from the data presented in Table 5, the modeling resulted in significant neurodegenerative damage to the rat brain. After modeling the pathology, the activity of the neuronal membrane integrity marker NSE in the control group significantly increased by 40 times compared to intact values, indicating the development of significant neuronal destruction and the prevalence of necrotic death.

By the end of the experiment, the level of another marker called the S-100 protein, which reflects the activity of astrocytic glia—a change that is a natural response of nervous tissue to necrotic and necrobiotic processes—increased 11-fold compared to intact values. The data obtained indicate a significant primary lesion of the neuronal array and intensification of neuroglial proliferative processes in the modeling of EAE.

Course administration of methylprednisolone to rats with EAE did not affect the level of neurospecific markers. Co-administration of methylprednisolone with mexidol led to a significant decrease in NSE by 22% and S-100 by 29.3% compared to the control group. It is worth noting that in the group of EAE rats treated with methylprednisolone and mexidol, the levels of NSE and S-100 were significantly lower than in the group treated with methylprednisolone alone. 

Course administration of catechin and methylprednisolone leads to a decrease in neurodegradation markers in the cytosol of rats with EAE: NSE by 37% and S-100 by 54.8%. In terms of the degree of NSE reduction, the combined administration of methylprednisolone significantly outperforms the combination of methylprednisolone with the reference drug mexidol. The results obtained indicate a significant neuroprotective effect of the ocular combination of methylprednisolone and catechin. The above confirms the correctness of the choice of bioflavonoid using a virtual screening program.

As can be seen from Table 6, the modeling of EAE significantly activates oxidative stress reactions by the end of the experiment as evidenced by an 11.25-fold increase in nitrotyrosine in the rat brain cytosol. Also, modeling of EAE leads to a significant surge in the neuroinflammatory reactions in response to the development of significant neurodegeneration and neuronal death by necrosis. Thus, we have noted a 12.5-fold increase in the proinflammatory cytokine IL-1b in the cytosol of brain homogenate of rats with EAE. IL-1b is produced by microglia, participates in the local inflammatory response around the necrosis “core” zone, and plays an important role in the mechanisms of secondary neuronal damage. Nuclear transcription factors AP-1 and NF-kB are activated when IL-1b interacts with receptors. This alters target cell behavior and triggers the development of an acute-phase cellular response, proinflammatory factor expression, astrocyte stimulation of iNOS and cytotoxic NO derivatives, increased permeability of mitochondrial pores, and neuroapoptosis [36].

Course administration of methylprednisolone significantly reduced the level of oxidative stress marker nitrotyrosine by 18% and inflammatory marker IL-1b by 63%. The addition of mexidol to methylprednisolone therapy led to a significant increase in the antioxidant effect—a 46.2% decrease in nitrotyrosine compared to the control and a 34% decrease in nitrotyrosine compared to the group receiving methylprednisolone alone. At the same time, the combination of methylprednisolone with mexidol did not affect the increase in anti-inflammatory effect. The level of IL-1b did not differ in both groups. The course administration of methylprednisolone with catechin to rats with EAE significantly increased both the antioxidant and anti-inflammatory effects of methylprednisolone. 

Thus, in the cytosol of the brain homogenate of rats with EAE treated with a combination of catechin, the level of nitrtyrosine was 58.4% lower than the control value, and IL-1b was 74.2% lower. The values of these markers in the group receiving the combination with catechin were significantly lower than in the group with methylprednisolone monotherapy and methylprednisolone and the reference drug mexidol. This can be explained from the standpoint of the antioxidant mechanism of catechin action, which regulates the level of reactive oxygen species (ROS) and, thus, is able to regulate the expression of proinflammatory cytokines [37]. This can also be explained from the point of view that catechin is able to inhibit the formation of NO. This is evidenced by both the results of the virtual screening program and the results of the in vitro experiments on inhibition of photoinduced NO formation. The reduction in IL-1b under the influence of catechin and the enhancement of this effect when it is administered together with methylprednisolone can be explained by its binding to transthyretin, which plays an important role in the development of neuroinflammation [10].

## 3. Materials and Methods

### 3.1. Docking Analysis

The docking analysis was the initial stage. Flexible molecular docking was used in this study to identify compounds with an affinity for a particular biological target. Protein Data Bank (PDB) macromolecules, namely human transthyretin in combination with thyroxine (T4), were utilized as a biological target. (PDB ID—1ICT) [Protein Data Bank. http://www.rcsb.org/pdb/home/home.-do. Accessed 6 September 2021]. 

Ligand preparation: Substances were drawn using MarvinSketch 20.20.0 and saved in mol format [MarvinSketch version 20.20.0, ChemAxon http://www.chemaxon.com Accessed 20 April 2021]. After initial preparation, the ligands were optimized using Chem3D with the molecular mechanics algorithm MM2 and saved as pdb files. Molecular mechanics was employed to obtain more realistic geometric values for most organic molecules due to its high parameterization. Using AutoDockTools-1.5.6, the pdb files were converted to PDBQT format, with the number of active torsions set to default.

Protein preparation: Protein structures were downloaded from the Protein Data Bank. Discovery Studio v19.1.0.18287 was used to remove water molecules and ligands from the protein structures, which were then saved as pdb files [Discovery Studio Visualizer v19.1.0.18287, Accelrys Software Inc., San Diego, CA, USA, https://www.3dsbiovia.com Accessed 20 April 2021]. In AutoDockTools 1.5.6, polar hydrogen atoms were added to the protein structures, which were then saved as PDBQT files. The grid field was set as follows: center_x = −1.750, center_y = −44.944, center_z = 33.278, size_x = 25, size_y = 25, size_z = 25 for the receptor (PDB ID—1ICT). Vina was used for docking. Discovery Studio v 19.1.0.18287 was used for visualization.

### 3.2. The Virtual Screening Program

The following machine learning models can be used for a virtual screening program:

1. Linear Regression

Linear Regression is a machine learning method that uses a linear model to predict numerical values. It finds a linear relationship between input data and output values that allows you to make a prediction for new data.

2. Support Vector Machine Regression

Support vector machine regression is a machine learning method used for regression tasks. It finds the hyperplane in the input space that separates the values of the output variable the most. The new input data can then be used to predict the output value.

3. Random Forest Regression

Random Forest Regression is a machine learning method used for regression tasks. It creates several different random forest trees, each of which predicts the value of the output variable. The predictions of each tree are then combined to obtain the final result.

4. Gradient Boosting Regression

Gradient boosting regression is a machine learning method used for regression tasks. It creates successive models, each of which corrects the errors of the previous model. The predictions of each model are then combined to produce the final result.

5. K-Nearest Neighbors Regression

The k-nearest neighbors (KNN) method is one of the machine learning algorithms used for classification and regression. This method searches for the k-nearest points (neighbors) to the point under study in the input data space and uses their values to predict the output variable for the point under study.

**Feature scaling**

Before building the models, we need to scale the features. While linear regression and random forest algorithms do not require this procedure, other methods, such as k-nearest neighbors, do, as they rely on the calculation of Euclidean distance. 

For our purposes, we will use the MinMaxScaler.

# Create the scaler object with a range of 0–1

scaler = MinMaxScaler(feature_range = (0, 1))

# Fit on the training data

scaler.fit(train_features)

# Transform both the training and testing data

train_features = scaler.transform(train_features)

test_features = scaler.transform(test_features)

**Evaluation of different models**

We will prepare a number of auxiliary functions for running models and evaluating them.

# Function to calculate mean absolute error

def mae(true_labels, predicted_labels):

   return mean_absolute_error(true_labels, predicted_labels)

# Takes in a model, trains the model, and evaluates the model on the test set

def fit_and_evaluate(model, train_features, train_labels, test_features, test_labels):

# Train the model

   model.fit(train_features, train_labels)

# Make predictions and evalute

   model_test_pred = model.predict(test_features)

   model_train_pred = model.predict(train_features)

   model_test_mae = mae(test_labels, model_test_pred)

   model_train_mae = mae(train_labels, model_train_pred)

# Return the performance metric

   return model_test_mae, model_train_mae

#### 3.2.1. Linear Regression Model

This model’s output displays an error of 16% on the training sample and 20% on the test sample, respectively.

lr = LinearRegression()

lr_mae_test, lr_mae_train = fit_and_evaluate(lr, train_features, train_labels, test_features, test_labels)

print(‘Linear Regression Performance on the test set: MAE = %0.4f’ % lr_mae_test)

print(‘Linear Regression Performance on the train set: MAE = %0.4f’ % lr_mae_train)

Linear Regression Performance on the test set: MAE = 20.0076

Linear Regression Performance on the train set: MAE = 16.0950

#### 3.2.2. Regression Model Using the Support Vector Method

The result of this model displays an error of 18.97% on the test sample and 17.47% on the training sample, respectively.

svm = SVR()

svm_mae_test, svm_mae_train = fit_and_evaluate(svm, train_features, train_labels, test_features, test_labels)

print(‘Support Vector Machine Regression Performance on the test set: 

MAE = %0.4f’ % svm_mae_test)

print(‘Support Vector Machine Regression Performance on the train set: 

MAE = %0.4f’ % svm_mae_train)

Support Vector Machine Regression Performance on the test set: MAE = 18.9718

Support Vector Machine Regression Performance on the train set: MAE = 17.4767

#### 3.2.3. Random Forest Model

The result of this model displays an error of 23.72% on the test sample and 6.94% on the training sample, respectively.

random_forest = RandomForestRegressor(random_state=0)

random_forest_mae_test, random_forest_mae_train = fit_and_evaluate(random_forest, train_features, train_labels, test_features, test_labels)

print(‘Random Forest Regression Performance on the test set: MAE = %0.4f’ % random_forest_mae_test)

print(‘Random Forest Regression Performance on the train set: MAE = %0.4f’ % random_forest_mae_train)

Random Forest Regression Performance on the test set: MAE = 23.7254

Random Forest Regression Performance on the train set: MAE = 6.9407

#### 3.2.4. Gradient Boosting Model

The result of this model displays an error of 21.98% on the test sample and 3.6% on the training sample, respectively.

gradient_boosted = GradientBoostingRegressor(random_state = 0)

gradient_boosted_mae_test, gradient_boosted_mae_train = fit_and_evaluate(gradient_boosted, train_features, train_labels, test_features, test_labels)

print(‘Gradient Boosted Regression Performance on the test set: MAE = %0.4f’ % gradient_boosted_mae_test)

print(‘Gradient Boosted Regression Performance on the train set: MAE = %0.4f’ % gradient_boosted_mae_train)

Gradient Boosted Regression Performance on the test set: MAE = 21.9783

Gradient Boosted Regression Performance on the train set: MAE = 3.5952

#### 3.2.5. K-Nearest Neighbors Model

The result of this model displays an error of 18.78% on the test sample and 14.21% on the training sample, respectively.

knn = KNeighborsRegressor()

knn_mae_test, knn_mae_train = fit_and_evaluate(knn, train_features, train_labels, test_features, test_labels)

print(‘K-Nearest Neighbors Regression Performance on the test set: MAE = %0.4f’ % knn_mae_test)

print(‘K-Nearest Neighbors Regression Performance on the train set: 

MAE = %0.4f’ % knn_mae_train)

K-Nearest Neighbors Regression Performance on the test set: MAE = 18.7883

K-Nearest Neighbors Regression Performance on the train set: MAE = 14.2183

The graph below demonstrates that the support vector and k-nearest neighbors models show similar results on the test sample and demonstrate the smallest error (about 18%).

plt.style.use(‘fivethirtyeight’)

# Dataframe to hold the results

model_comparison = pd.DataFrame({‘model’: [‘Linear Regression’, ‘Support Vector Machine’,

‘Random Forest’, ‘Gradient Boosted’,

‘K-Nearest Neighbors’],

‘mae_test’: [lr_mae_test, svm_mae_test, random_forest_mae_test,

gradient_boosted_mae_test, knn_mae_test],

‘mae_diff’: [lr_mae_test − lr_mae_train, svm_mae_test − svm_mae_train, random_forest_mae_test − random_forest_mae_train,

gradient_boosted_mae_test − gradient_boosted_mae_train, knn_mae_test − knn_mae_train]

})

# Horizontal bar chart of test mae

model_comparison.sort_values(‘mae_test’, ascending = False).plot(x = ‘model’, y = ‘mae_test’, kind = ‘barh’,

color = ‘red’, edgecolor = ‘black’)

# Plot formatting

**Figure 1:**
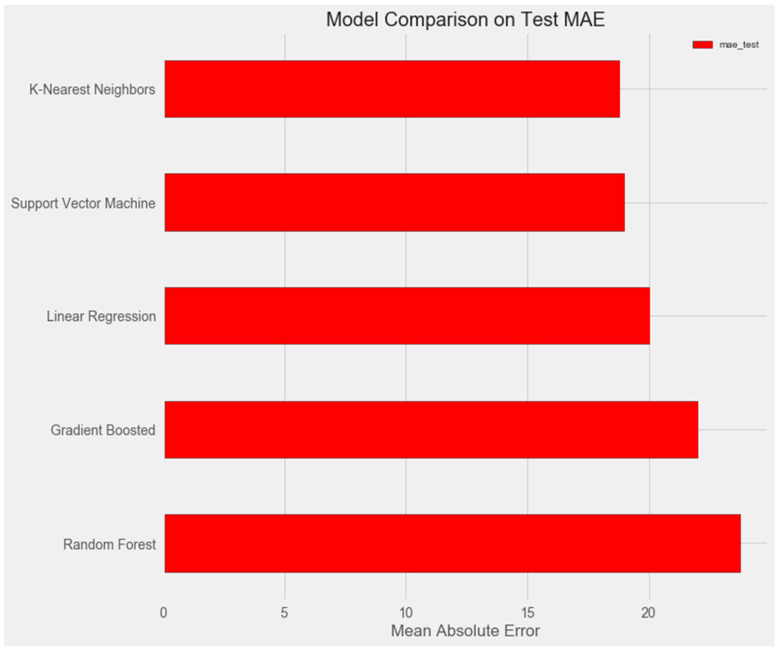
Comparison of models on the test set.

**Figure 2:**
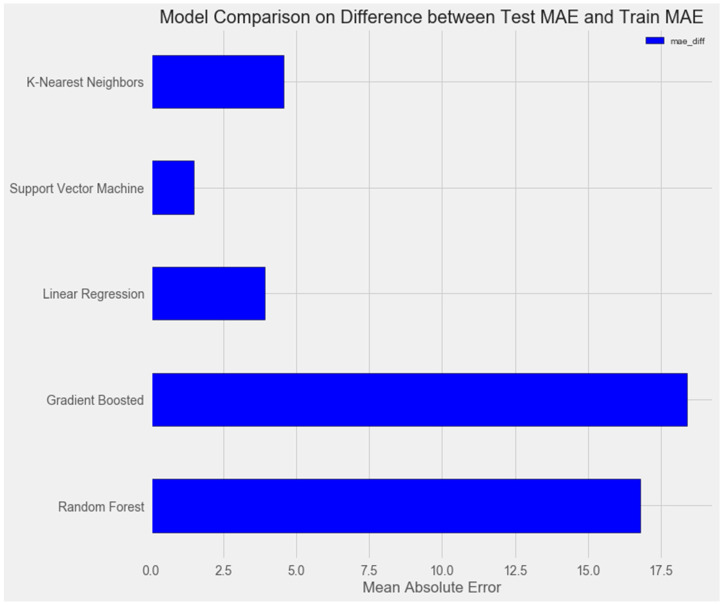
Comparison of models on the training set.

plt.ylabel(‘‘); plt.yticks(size = 14); plt.xlabel(‘Mean Absolute Error’); plt.xticks(size = 14)

plt.title(‘Model Comparison on Test MAE’, size = 20);

Upon comparing the error differences between the training and test samples, we observe that the “support vector” model exhibits the greatest generalization ability, while both the “random forest” and “gradient boosting method” models display clear signs of overfitting (Figure 1 and Figure 2).

# Horizontal bar chart of test mae

model_comparison.sort_values(‘mae_test’, ascending = False).plot(x = ‘model’, 

y = ‘mae_diff’, kind = ‘barh’,

color = ‘blue’, edgecolor = ‘black’)

# Plot formatting

Plt.ylabel(‘’); plt.yticks(size = 14); plt.xlabel(‘Mean Absolute Error’); 

plt.xticks(size = 14)

plt.title(‘Model Comparison on Difference between Test MAE and Train MAE’, size = 20);

Following this, we proceed to optimize the models and visualize their performance.

**Support vector method**

**Figure 3:**
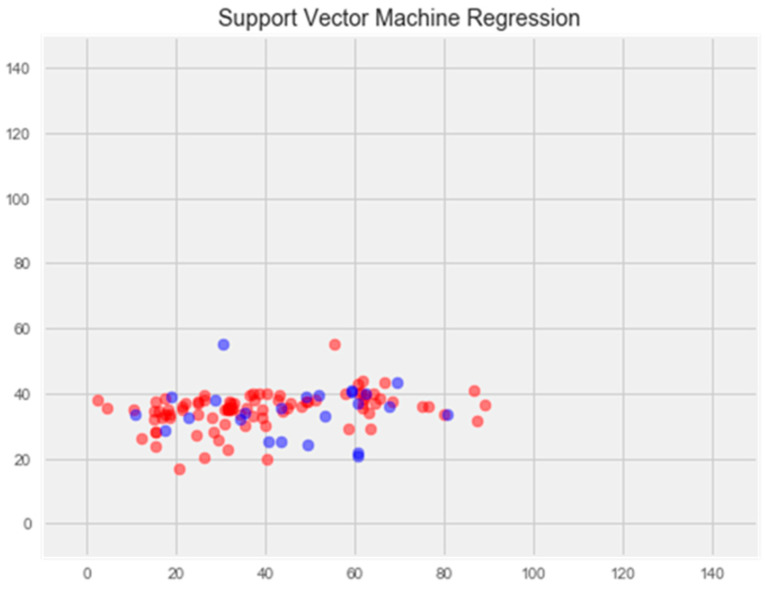
Optimization of the support vector model. **Note:** red color—test set, blue color—training set.

The optimization of the model resulted in an enhancement of prediction accuracy on the training set; however, this improvement was accompanied by a decrease in performance on the test set (Figure 3).

**R** **andom forest model**

**Figure 4:**
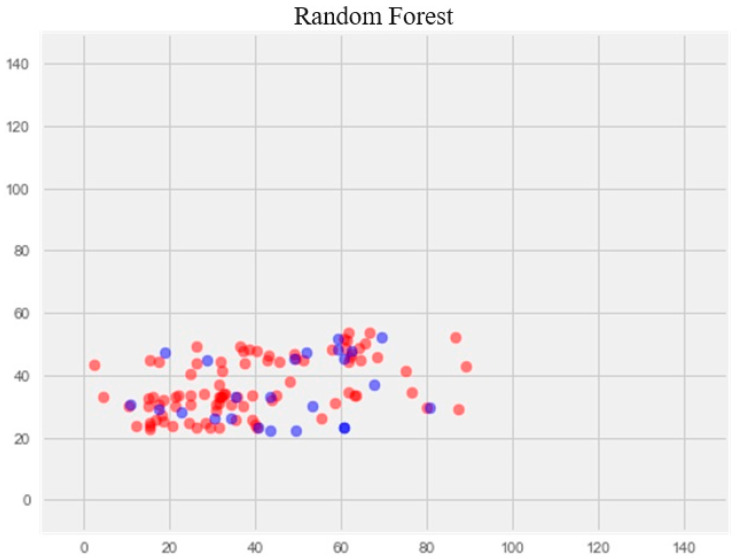
Optimization of the random forest model. **Note:** red color—test set, blue color—training set.

The optimization of the model resulted in a notable enhancement of generalization ability and effectively eliminated overfitting (Figure 4). The error rate on the training set was 14.77%, and on the test set, 17.69%.

**K-nearest neighbors model**

**Figure 5:**
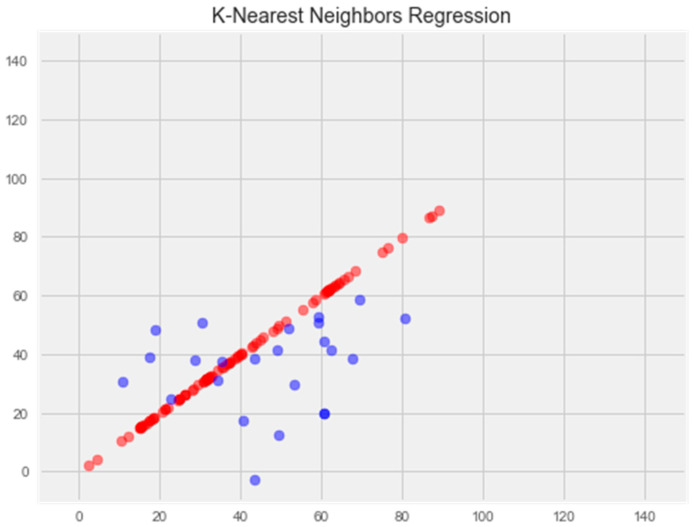
Optimization of the k-nearest neighbors model. **Note:** red color—test set, blue color—training set.

Optimization of the model led to overtraining and loss of generalization properties (Figure 5).

**Gradient boosting model**

**Figure 6:**
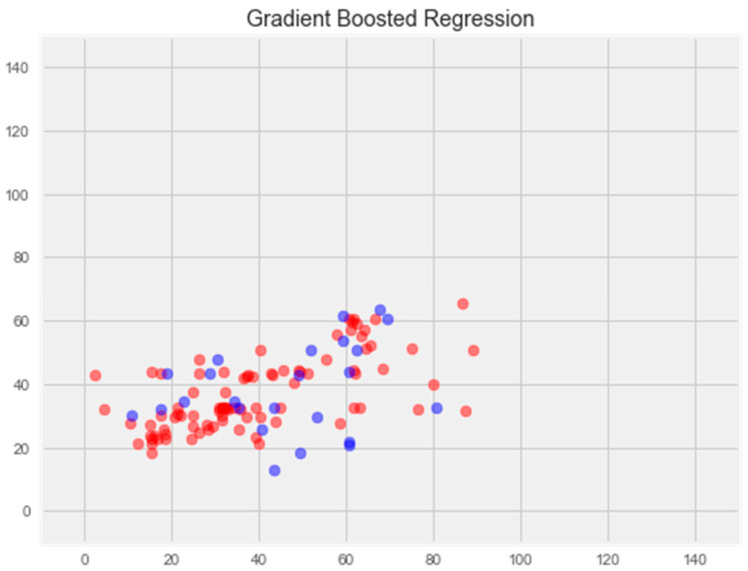
Optimization of the gradient boosting model. **Note:** red color—test set, blue color—training set.

Optimization of this model led to a significant improvement in the generalization ability and eliminated overfitting (Figure 6). The error rate on the training sample was 11.81%, and on the test sample was 16.65%, which is the best indicator among all models.

We explored a number of models for regress problem resolution after our investigation. Without optimization, the support vector machine (SVM) and k-nearest neighbors (KNN) models outperformed the others. Following model optimization, the gradient boosting model showed the greatest ability to generate new data, with an error rate of 16%. Based on quantum chemistry characteristics, antioxidant activity can be predicted using this improved model. 

**Figure 7:**
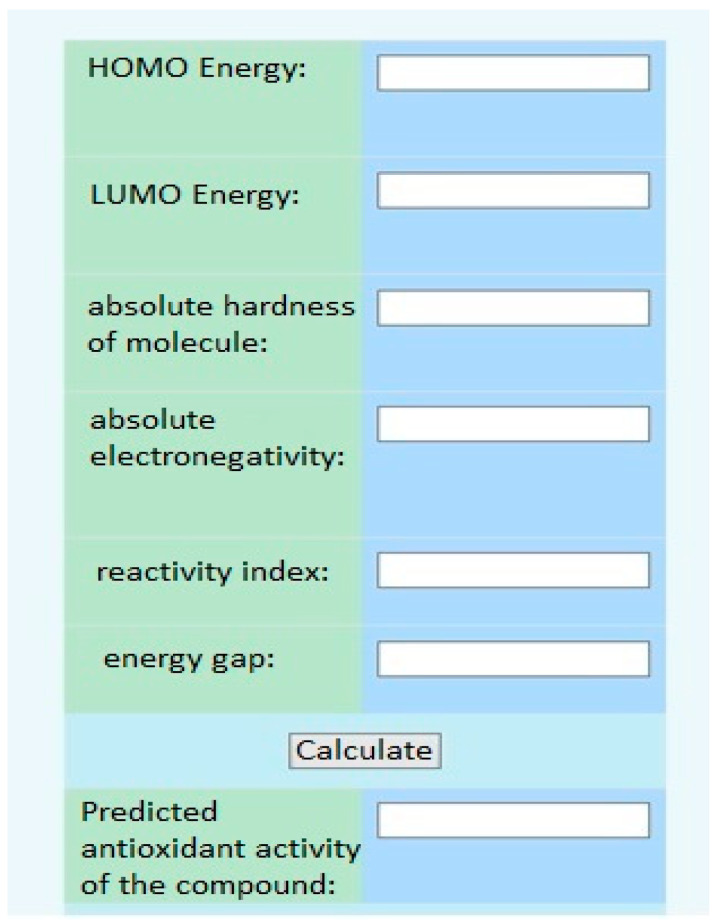
The interface of the virtual screening program.

Furthermore, using the electronic topological approach, the Department of Medical and Pharmaceutical Informatics and New Technologies at ZSMPU developed a virtual screening program (Figure 7).

### 3.3. In Vitro Studies

The second step involved spectrophotometric analysis to measure the displacement of T4 flavonoids from their complex with the TTR protein [37,38]. These experiments were carried out using a Libra S 32 PC spectrophotometer (Biochrome Ltd., Cambridge, UK). The TTR protein subunit had a purity of ≥95% and was sourced from LEE Biosolutions (Maryland Heights, MO, USA). The flavonoids and L-thyroxine substrates were of analytical grade with a purity of >95%, obtained from Sigma-Aldrich (Burlington, MA, USA). The binding studies were conducted in a solution containing 50 mM phosphate buffer at pH 7.4 and a temperature of 25 °C. The interaction between T4 + TTR complexes and bioflavonoids was assessed by monitoring the increase in optical density at 280 nm (indicative of protein binding) and at 225 nm (indicating the level of displaced thyroxine). All tested bioflavonoid samples exhibited maximum absorption at 280 nm. A thyroxine hormone T4 solution (20 nM/mL in 10 mM sodium hydroxide) was prepared in advance. Test samples were dissolved in phosphate buffer, resulting in a final flavonoid concentration of 0.05 μmol in each sample. The protein was also dissolved in phosphate buffer, with a final concentration of 0.1 μmol per sample. The T4 solution (final concentration of 20 nM) was added to recombinant TTR in phosphate buffer and incubated at 37 °C for 5 min. Subsequently, test samples were added to the incubation mixture and the volume was adjusted to 1.0 mL with phosphate buffer. The optical density was measured at two wavelengths. Following the initial optical density measurement, the samples were incubated in cuvettes at 37 °C for 25 min, after which the optical density was measured again. Each series (control and catechin samples) consisted of 10 samples analyzed spectrophotometrically.

The antioxidant activity (AOA) of bioflavonoids was also evaluated on the inhibition of NO radicals [18]. The method is based on the photoinduction of sodium nitroprusside, which results in the accumulation of NO radicals. The rate of ascorbate oxidation, assessed by measuring the optical density at 265 nm, indicates the presence of these radicals.

Study procedure: Immediately before the experiment, a 0.08% ascorbic acid solution and a 0.6% sodium nitroprusside solution were prepared. To start the reaction, 2 mL of the sodium nitroprusside solution was mixed with 1 mL of ascorbic acid and 0.5 mL of the test sample (concentration = 10^−6^ M). The mixture was then irradiated with a 300 W light source at a wavelength of λ = 425 nm for 30 min. The rate of ascorbate oxidation, which was inhibited by the presence of reactive oxygen species (ROS), was used to evaluate the reaction. The concentration of ascorbate was determined by measuring the optical density at λ = 265 nm.

The antioxidant activity (*AOA*) was calculated using the following formula:

$$AOA=\frac{D_{k}-D_{o}}{D_{k}}\cdot 100\%$$

where *D_k_* represents the optical density of the samples without any additives, *D_o_* represents the optical density of the test sample.

### 3.4. Experimental Model of Multiple Sclerosis

A total of 50 white outbred rats of both sexes, weighing 220–260 g, were used in the experiment. They were taken from the nursery of the National Academy of Medical Sciences of Ukraine’s Institute of Pharmacology and Toxicology. All animals were placed in quarantine (acclimatization) for a duration of 14 days. Every animal under quarantine had a daily behavior and general health examination, and their cages were checked twice a day for morbidity and death. Animals that satisfied the requirements to be included in the experiment were split into groups using the randomization procedure prior to this study’s commencement. During quarantine, animals that did not fit the requirements were not allowed to participate in the study. Animal cages were situated in different rooms. The light regime consisted of twelve hours of light and twelve hours of darkness. A constant air temperature of 19–25 °C and 50–70% relative humidity were maintained. Every day, the humidity and air temperature were measured. About fifteen volumes of air were given every hour by the ventilation regime that was set up. The experimental animals were housed in a typical vivarium with identical diets. The rats were kept in conventional cages with five individuals in each. The diet consisted of bread, root vegetables (carrots and beets), and fodder grains.

Experimental allergic encephalomyelitis (EAE) was induced by a single subcutaneous injection of an encephalitogenic mixture (EMS) in complete Freund’s adjuvant (CFA). The injection mixture per animal contained 100 mg of homologous spinal cord homogenate, 0.2 mL of CFA (5 mg/mL of killed mycobacteria), and 0.2 mL of saline. EMS was injected at the base of the tail under light ether anesthesia at a volume of 0.4 mL. The formation of EAE in animals was assessed by the development of neurological disorders, determined by the clinical and cumulative EAE index. The clinical index was determined by the following scale: muscle weakness of one limb—½ point, paresis—1 point, paralysis—1 ½ points. If multiple limbs were involved, the scores were summed. The absence of disorders was scored as 0 points, and death was scored as 6 points. Animals with a clinical index of ½–2 ½ points were considered to have a mild form of EAE, while scores of 3–6 points corresponded to a severe course of EAE [39].

### 3.5. Drugs and Doses

In vitro and in vivo studies included test samples of bioflavonoids: quercetin, catechin, epicatechin gallate, epicatechin, procyanidin B1, procyanidin B2, procyanidin B3, and catechin-3-gallate (Sigma, USA).

In the in vivo experiments, there were five groups of animals:

(1) Intact (10 rats);
(2) Control—untreated with EAE, received physiological saline (10 rats);
(3) Animals with EAE receiving baseline treatment—methylprednisolone (MP) at 3.4 mg/kg, administered intraperitoneally, slowly, in saline (10 rats);
(4) Animals with EAE receiving MP + catechin at 10 mg/kg, administered intragastrically (10 rats);
(5) Animals with EAE receiving MP + antioxidant mexidol at 250 mg/kg, administered intragastrically (10 rats).

Two days following the induction of EAE, the following medications were given: methylprednisolone for seven days, and mexidol and catechin for fourteen days (latent phase plus clinical phase until the end of the disease’s peak). Throughout the course of treatment, intraperitoneal and intragastric saline were administered in comparable amounts to the control and intact rats. Day 17 of the trial was the final date of all studies.

Animal Euthanasia. At the end of the experiment, the animals were euthanized under thiopental sodium anesthesia (40 mg/kg).

### 3.6. Preparation of Biological Material

The brain’s blood supply was quickly cut off, and the dura mater was detached from the brain. After being submerged in liquid nitrogen, the brain portions under investigation were crushed into a powder and homogenized in a 10-fold volume of media at 2 °C that contained (in mmol): 1 EDTA, 20 Tris–HCl buffer, and 250 sucrose (pH 7.4). Using a Sigma 3-30k refrigerated centrifuge (Sigma, Osterode am Harz, Germany), the mitochondrial fraction was separated by differential centrifugation at +4 °C. The homogenate was spun for 7 min at 1000× *g* to separate the mitochondrial fraction from large cellular pieces. The supernatant was then centrifuged again for 20 min at 17,000× *g*. After being decanted, the supernatant was kept cold at −80 °C.

### 3.7. Enzyme-Linked Immunosorbent Assay

The cerebral cytosol was used to determine neurodegeneration markers, such as neuron-specific enolase (NSE) and protein S100 (S100), which reflect neuroglial activity as a natural response to massive neuronal destruction. NSE activity (ng/mL) and S100 content (ng/mL) were determined by enzyme-linked immunosorbent assay (ELISA) using the NSE ELISA KIT #MBS2024030 (MyBioSource, Inc., San Diego, CA, USA) and S-100 ELISA KIT #LS-F25201 (LifeSpan Biosciences, Inc., Shirley, MA, USA).

IL-1β was determined as an inflammatory marker involved in the mechanisms of secondary brain damage. The significance of this marker was confirmed by studies that revealed a correlation between increased IL-1β levels, the severity of neurological disorders, and growth factor deficiency (BDNF, IGF-1, PDGF). IL-1β was determined in the retinal homogenate by ELISA using the IL-1β ELISA KIT #KE10003 (Proteintech, Rosemont, IL, USA).

As a marker of oxidative stress and to assess the antioxidant effect of the studied drugs, nitrotyrosine was determined by solid-phase enzyme immunoassay using the NITROTYROSINE ELISA KIT #HK501 (HycultBiotech, Wayne, PA, USA). This study was carried out using a Sirio-S microplate immunoassay reader (Seac Radim Company, Roma, Italy).

### 3.8. Statistical Methods of this Study

The standard statistical package of the licensed software “STATISTICA^®^ for Windows 6.0” (StatSoft Inc. Hamburg, Germany, № AXXR712D833214FAN5), “SPSS 16.0”, and “Microsoft Office Excel 2003” were used to calculate the study’s results. The Shapiro–Wilk test was utilized to determine whether the distribution was normal. The mean values of the data were sent beforehand. Student’s *t*-test, for a normal distribution, was used to assess the dependability of the mean value variations. The Mann–Whitney U test was employed when analyzing ordinal variables or when there was a non-normal distribution. Analysis of variance (ANOVA) was employed for normal distributions and the Kruskal–Wallis test for non-normal distributions when comparing independent variables in more than two samples. Differences with *p* < 0.05 (95%) were deemed statistically significant for all methods of analysis.

Quantum chemical calculations: in the course of this study, quantum chemical calculations of the descriptors HOMOEnergy (highest occupied molecular orbital) and LUMOEnergy (lowest vacant molecular orbital) were performed using the WinMopac software package (v. 7.2). The structure was optimized using the semi-empirical AM1 method with the following parameters: Calculation = SinglePoint, WaveFunction = ClosedShell (RHF).

## 4. Conclusions

As a result of using new information technologies, we have selected a catechin from a large number of bioflavonoids that according to the results of docking analysis can form complexes with transthyretin, a protein involved in the pathogenesis of MS, and can also be a potential scavenger of NO, which is also involved in the pathogenesis of MS. Thus, a computer program for virtual screening showed a 55% prediction. In vitro studies have confirmed the high potential of catechin as a neuroprotective treatment for MS.

## Figures and Tables

**Table 1:** Molecular docking analysis of the affinity of the test samples and reference products for human transthyretin (TTR).

Compound	Affinity (kcal/mol) to Human Transthyretin	Docking 2D Visualization	
Thyroxin	−6.4	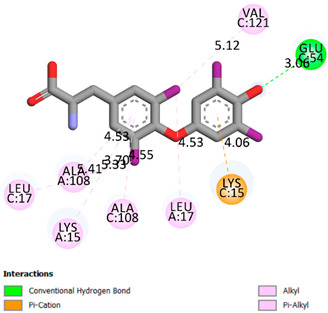	
Quercetin	−6.7	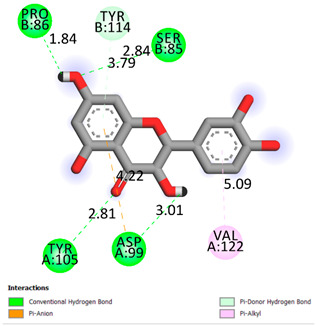	
Catechin	−6.2	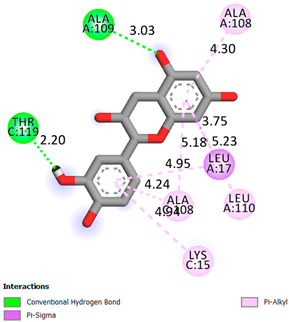	
Epicatechin	−7.2	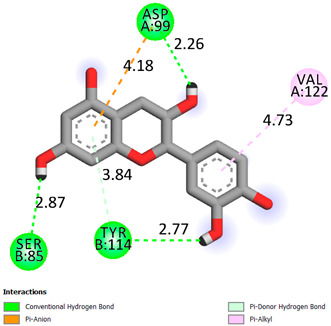	
Catechin-3-gallate	−7.6	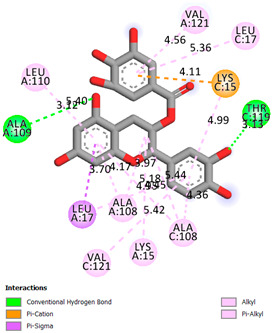	
Epicatechin-3-gallate	−7.3	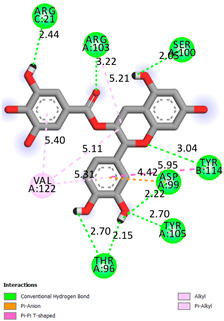	
Epigallocatechin 3-O-Gallate	−7.7	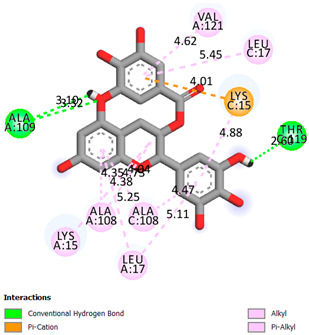	
Gallocatechin-3-gallate	−7.5	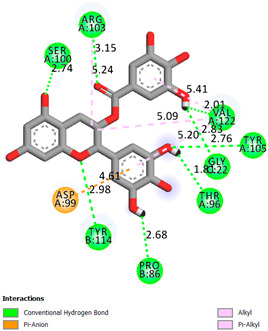	
Kaempferol	−7.2	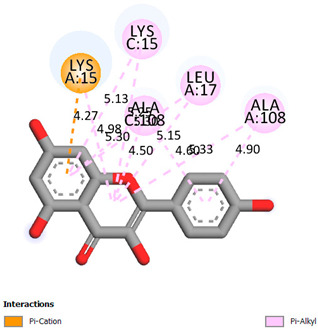	
Luteolin	−8.0	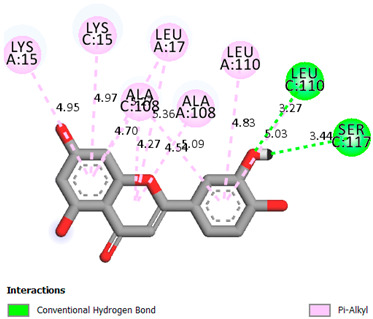	
Procyanidin B1	−7.4	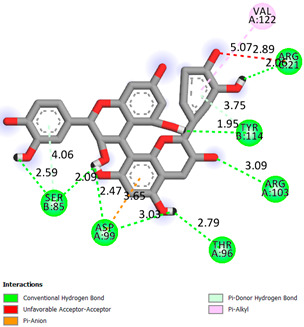	
Procyanidin B2	−8.6	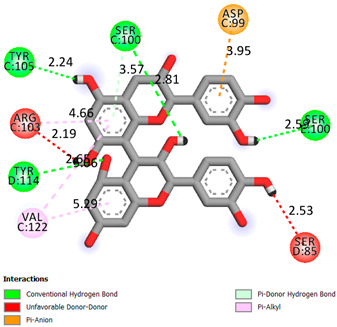	
Procyanidin B3	−8.7	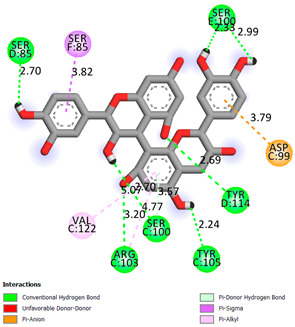	

**Table 2:** Results of UV spectrophotometry of Transthyretin + L-Thyroxine samples after addition of flavonoid samples and incubation for 25 min at 37 °C.

Experimental Series	Variation in Optical Density at 280 nm (Protein Binding)	Variation in Optical Density at 225 nm (Level of Displaced Thyroxine)
Indicators	∆	∆
Control (incubation mixture without catechin)	0 ± 0	0 ± 0
Experimental (incubation mixture containing catechin)	0.063 ± 0.0001 **	0.082 ± 0.0002 **

Notes: 1. Data are presented as “M ± m”, where M represents the mean value and m denotes the standard error of the mean. 2. “∆” indicates the change in optical absorbance before and after incubation. 3. “**” signifies a *p*-value of <0.001.

**Table 3:** Computational prediction with the help of a program using the “gradient booster model and results of the in vitro antioxidant activity of catechin on NO inhibition.

Code	AOA Results at 10^−6^ M		AOA Prediction, %.
	E, M ± m	%	
Catechin	1.625 ± 0.001	43.67	55.15
Control	1.131 ± 0.002 **	-	

Notes: 1. Data are presented as “M ± m”, where M represents the mean value and m denotes the standard error of the mean. 2. “**” signifies a *p*-value of <0.001.

**Table 4:** Effect of catechin on the course of EAE in the setting of baseline methylprednisolone therapy.

Indicators	Groups of Animals			
	Control, EAE (*n* = 10)	MP (*n* = 10)	Catechin + MP (*n* = 10)	Mexidol + MP (*n* = 10)
% of sick animals (total/severe)	100/70	80/30	80/10 *^1^	80/20 *
Average clinic index at the peak of EAE, points	2.6 + 0.5	1.80 + 0.5	0.9 + 0.5 *^1^	1.65 + 0.152
Average cumulative index, points	27.2 + 1.5	9.4 + 0.4 *	6.2 + 0.4 *^1^	7.5 + 0.6 *
Duration of EAE, days (Student’s test)	16.0 + 1.2	8.4 + 0.7 *	6.4 + 0.2 *^1^	7.2 + 0.8 *

Notes. *—*p* ≤ 0.05 in relation to the control values; ^1^—*p* ≤ 0.05 compared to methylprednisolone.

**Table 5:** Concentration of neurodegradation markers—neuron-specific enolase (NSE) and protein S-100—in the cytosol of rats with EAE after experimental therapy.

Experimental Groups	NSE, ng/mL	S-100, ng/mL
Intact (n = 10)	0.223 ± 0.015	0.088 ± 0.002
EAE (control) (n = 10)	9.11 ± 0.15 ^1^	0.97 ± 0.015 ^1^
MP (n = 10)	9.15 ± 0.14 ^1^	0.92 ± 0.033 ^1^
MP+ Mexidol (n = 10)	7.11 ± 0.21 *^1,2^	0.65 ± 0.042 *^1,2^
MP+ catechin (n = 10)	5.74 ± 0.11 *^1,2,3^	0.438 ± 0.014 *^1,2,3^

Note: *—*p* < 0.05 compared to the control group; ^1^—*p* < 0.05 in relation to the intact group; ^2^—*p* < 0.05 in relation to the MP group; ^3^—*p* < 0.05 in relation to the mexidol group.

**Table 6:** Concentration of inflammatory markers IL-1b and oxidative stress nitrotyrosine in the cytosol of brain homogenate of rats with EAE and experimental therapy.

Experimental Groups	Nitrotyrosine, ng/mL	IL-1b, ng/mL
Intact (*n* = 10)	0.88 ± 0.042	0.31 ± 0.018
EAE (control) *(n* = 10)	9.89 ± 0.33 *^1^	3.88 ± 0.055 ^1^
MP (*n* = 10)	8.11 ± 0.40 ^1^	1.44 ± 0.022 *^1^
MP+ Mexidol (*n* = 10)	5.32 ± 0.32 *^1,2,^	1.39 ± 0.033 *^1,^
MP+ catechin (*n* = 10)	4.11 ± 0.07 *^1,2,3^	1.00 ± 0.02 *^2,3^

Note: *—*p* < 0.05 compared to the control group; ^1^—*p* < 0.05 in relation to the intact group; ^2^—*p* < 0.05 in relation to the MP group; ^3^—*p* < 0.05 in relation to the mexidol group.

## Data Availability

All the data generated during this research are included in the manuscript.
